# Efficacy and safety of adjuvant EGFR-TKIs for resected non-small cell lung cancer: a systematic review and meta-analysis based on randomized control trials

**DOI:** 10.1186/s12885-022-09444-0

**Published:** 2022-03-26

**Authors:** Pengfei Zhao, Hongchao Zhen, Hong Zhao, Lei Zhao, Bangwei Cao

**Affiliations:** 1 Department of Radiotherapy, Beijing Friendship Hospital, Capital Medical University, No.95 Yong An Road, Xicheng District, Beijing, 100050 China; 2 Department of Oncology, Beijing Friendship Hospital, Capital Medical University, No.95 Yong An Road, Xicheng District, Beijing, 100050 China

**Keywords:** Resected tumor, Adjuvant EGFR-TKI, NSCLC, Adjuvant therapy, Adjuvant chemotherapy

## Abstract

**Background:**

Postoperative adjuvant cisplatin-based chemotherapy had been the standard care in patients with completely resected high-risk stage IB to IIIA non-small cell lung cancer (NSCLC) for decades. However, the survival benefits were far from satisfactory in clinical practice. Thus, this meta-analysis was performed to compare the efficacy and safety of adjuvant epidermal growth factor receptor tyrosine kinase inhibitors (EGFR-TKIs) in patients with resected NSCLC based on updated literature and research.

**Methods:**

A systematic literature search based on random control trials (RCTs) was conducted with keywords on PubMed, Embase and the Cochrane library databases. All articles compared EGFR-TKIs to placebo or chemotherapy as adjuvant therapies for early-stage resected NSCLC. A meta-analysis was performed to generate combined hazard ratio (HR) with 95% confidence intervals (CI) for disease-free survival (DFS), overall survival (OS), and risk ratio (RR) with 95% CI for disease recurrence and adverse events (AEs). The Stata statistical software (version 14.0) was used to synthesis the data.

**Results:**

A total of 9 RCTs comprising 3098 patients were included. Adjuvant EGFR-TKIs could significantly prolong DFS in patient with resected NSCLC harboring epidermal growth factor receptor (*EGFR*) mutations (HR 0.46, 95% CI 0.29–0.72), but had no impact on OS (HR 0.87, 95% CI 0.69–1.11). The subgroup analyses indicated that adjuvant EGFR-TKIs were superior in regard to DFS in most subgroups, including varied smoking status, *EGFR* mutations type, gender, age, Eastern Cooperative Oncology Group performance status and adenocarcinoma. Osimertinib resulted in decreased brain recurrence than first generation of EGFR-TKIs (RR 0.12, 95% CI 0.04–0.34 vs. RR 1.07, 95% CI 0.64–1.78, respectively). The AEs were generally manageable and tolerable. The incidence of high-grade (≥ 3) AEs including diarrhea (RR 5.68, 95% CI 2.94–10.98) and rash (RR 27.74, 95% CI 11.43–67.30) increased after adjuvant EGFR-TKIs treatment.

**Conclusions:**

Adjuvant EGFR-TKIs therapy could significantly prolong DFS in patients with completely resected early-stage *EGFR* mutation-positive NSCLC, but had no impact on OS. Adjuvant EGFR-TKIs could be an important treatment option in patients with resected early-stage *EGFR*-mutant NSCLC.

**Supplementary Information:**

The online version contains supplementary material available at 10.1186/s12885-022-09444-0.

## Background

Lung cancer is considered as the leading cause of cancer-related mortality in the world [1]. Completed anatomical pulmonary resection and intrathoracic lymph node dissection with at least six stations of lymph nodes have been the most effective and preferred strategy in the treatment of early-stage (stage I-IIIA) non-small cell lung cancer (NSCLC). However, only 30% of patients with NSCLC are considered candidates for surgical resection at first diagnosed [2, 3]. Approximately 30–70% of patients will relapse and progress with metastases despite undergoing complete resection and adequate adjuvant treatment [4, 5]. Therefore, an effective adjuvant therapy is necessary to eliminate the microscopic residual lesions. According to the recommendations from previous studies and National Comprehensive Cancer Network (NCCN) guideline, postoperative adjuvant cisplatin-based chemotherapy has been the standard care in patients with completely resected high-risk stage IB and stage II-IIIA NSCLC irrespective of epidermal growth factor receptor (*EGFR*) mutation status for decades [6, 7]. However, only a 16% decrease in the risk of disease recurrence or death and a 5-year absolute survival benefit of 5.4% and 5-year disease-free survival (DFS) benefit of 5.8% are obtained with adjuvant chemotherapy [6–9]. A recent meta-analysis published in 2015 showed that DFS increased by just 4.0% with adjuvant chemotherapy relative to resection alone [2]. In general, comparison of these analyses suggests that the contribution of cisplatin-based adjuvant treatment has reached a therapeutic plateau and has been no substantial improvement in the overall outcomes during the past two decades. The prognosis of operable NSCLC is still far from satisfactory, at present. Further survival improvements should be sought through the use of alternative treatments with better tolerability than adjuvant chemotherapy.

*EGFR* mutation has a vital pathogenic and oncogenic role in NSCLC, which is observed in approximately up to 50% of patients with adenocarcinoma of lung in Asia. Epidermal growth factor receptor tyrosine kinase inhibitors (EGFR-TKIs), such as erlotinib [10], gefitinib [11] and osimertinib [12] are the recommended first-line treatments for advanced NSCLC harboring driver gene mutations (such as small multi-nucleotide in-frame deletions in exon 19 and a point mutation in exon 21 resulting in substitution of leucine for arginine at position 858 (L858R) of *EGFR*) [13, 14]. The effectiveness in response rates and significantly prolonged survival of EGFR-TKIs compared with doublet chemotherapy in advanced NSCLC have led to a series of studies involving EGFR-TKIs as an adjuvant treatment for resected NSCLC. A retrospective study indicated that adjuvant gefitinib could provide a significantly prolonged DFS compared to adjuvant chemotherapy in patients with completely resected *EGFR*-mutant stage II-IIIA NSCLC, which was 34.9 months versus 19.3 months [15]. Previous cohort study demonstrated that adjuvant erlotinib for 2 years after standard adjuvant chemotherapy with or without radiotherapy could improve the survival of patients with surgically resected *EGFR*-mutant stage IA-IIIA NSCLC, with a remarkable improved 2-year DFS greater than 85% [16]. Nevertheless, subsequent randomized controlled trials (RCTs) yielded conflicting results with respect to whether adjuvant EGFR-TKIs treatment compared to placebo or adjuvant chemotherapy could improve the prognosis of patients with operable NSCLC [17–24].

Three previous meta-analyses showed that therapy consisting of adjuvant EGFR-TKIs had specific advantage over placebo or adjuvant chemotherapy in terms of DFS for NSCLC patients with *EGFR* mutations undergoing complete resection, but the overall survival (OS) could not be synthesized because of immature follow-up data. However, adjuvant EGFR-TKIs had no survival benefit in patients without *EGFR* mutations [25–28]. EGFR-TKIs could be an alternative adjuvant treatment for patients who had undergone complete resection of histologically or pathologically confirmed early-stage NSCLC harboring *EGFR* mutations, with better tolerability and survival improvements than chemotherapy. So far, adjuvant EGFR-TKI of osimertinib has been considered to be recommended for resected NSCLC as an adjuvant treatment option by guidelines, but adjuvant cisplatin-based chemotherapy is still the preferred recommendation [29]. Thus, in order to further improve the treatment strategy and management of resected NSCLC, we performed this updated meta-analysis to summarize the efficacy and safety of adjuvant EGFR-TKIs for patients with resected NSCLC based on updated data and new evidence.

### Eligibility criteria

We included trials that met the following criteria in our meta-analysis: (1) Patients with completely resected, early-stage (stage I to III) pathological confirmed NSCLC; (2) Phase 2/3 RCTs comparing adjuvant EGFR-TKIs with chemotherapy or placebo; (3) Primary endpoints such as OS or DFS were reported; (4) Safety and adverse events (AEs) of EGFR-TKI or chemotherapy were evaluated in these trials. Only officially published English literature was included in the analysis.

### Literature research strategy

The meta-analysis was reported following the Preferred Reporting Items for Systematic Reviews and Meta-Analyses (PRISMA) statement [30]. Two researchers (Pengfei ZHAO and Hong ZHAO) separately searched PubMed, Embase and the Cochrane library databases for studies between January 1, 2010 and February 16, 2022 using common keywords related to adjuvant EGFR-TKI and resected NSCLC. The following keywords were included: “EGFR-TKI OR epidermal growth factor receptor tyrosine kinase inhibitors OR erlotinib OR gefitinib OR osimertinib OR icotinib OR dacomitinib OR afatinib” AND “lung neoplasms OR carcinoma, non-small-cell lung OR non-small cell lung cancer OR NSCLC OR resected NSCLC OR operable NSCLC” AND “adjuvant therapy”. Bibliographies of published articles and clinical trial registers were searched and reviewed for additional articles.

### Data extraction and quality evaluation

Two investigators (Pengfei ZHAO and Hongchao ZHEN) independently reviewed all the articles and extracted the data. The discrepancies were resolved by discussing with a third investigator until a consensus was reached. For individual study, trial name, authors’ last name, publication year, phase, country, study design, stage, number of patients in the EGFR-TKIs treatment and the control group, treatment regimes, percentage of *EGFR* mutations, percentage of receiving adjuvant chemotherapy, duration of EGFR-TKIs, follow-up, survival outcomes, adverse events and place of relapse were extracted carefully. Patients with early-stage NSCLC administering adjuvant EGFR-TKIs (sequential after chemotherapy or single used) after disease resection were defined as experimental group, and receiving adjuvant chemotherapy or placebo were as control group. The risk of bias tool (Cochrane Handbook for Systematic Reviews of Interventions) was used to assess the methodological quality of individual included studies, in which random sequence generation, allocation concealment, blinding of participants and personnel, blinding of outcome data, incomplete date, selective reporting and other bias were assessed [31, 32]. High risk, unclear risk and low risk were assessed and described in above-mentioned bias. The results were performed with risk of bias summary and risk of bias graph by using Review Manager 5.3 software (Cochrane Collaboration 2014, Nordic Cochrane Center, Copenhagen, Denmark).

### Statistical analysis

The Stata 14.0 statistical software (Stata Corporation, College Station, Texas, UAS) was used to conduct the meta-analysis. We chose DFS as the primary endpoint in this meta-analysis. DFS was defined as the time from randomization to disease recurrence or death from any cause. The other endpoints included OS, safety and toxicities and places of relapse. Hazards ratios (HR) with 95% confidence intervals (CI) was extracted from individual studies for survival outcome data. Risk ratio (RR) was estimated to represent the combined effect for dichotomous outcomes such as adverse events and places of relapse by extracting the number of events and the no occurred events in each group. Subgroup analyses were conducted based on variables such as smoking status, *EGFR* mutations type, histology, gender, age, Eastern Cooperative Oncology Group (ECOG) performance status, stage, receiving adjuvant chemotherapy or not, different EGFR-TKIs type. Heterogeneity analysis was performed by Chi-square test and χ^2^ *P* value < 0.1or an I^2^ statistic index > 50% indicated as statistical significance. A fixed-effects statistical model was used when there was no heterogeneity. Otherwise, a random-effects statistical model was applied. For safety and relapse, RR > 1 indicated that higher incidence of adverse events and higher recurrence occurred in patients treated with EGFR-TKIs than placebo or chemotherapy. The combined effects were confirmed statistically significant when *P* value < 0.05. Publication bias was assessed according to the Begg’s and Egger’s tests.

## Results

### Characteristic of the included studies and risk of bias

**Fig. 1:**
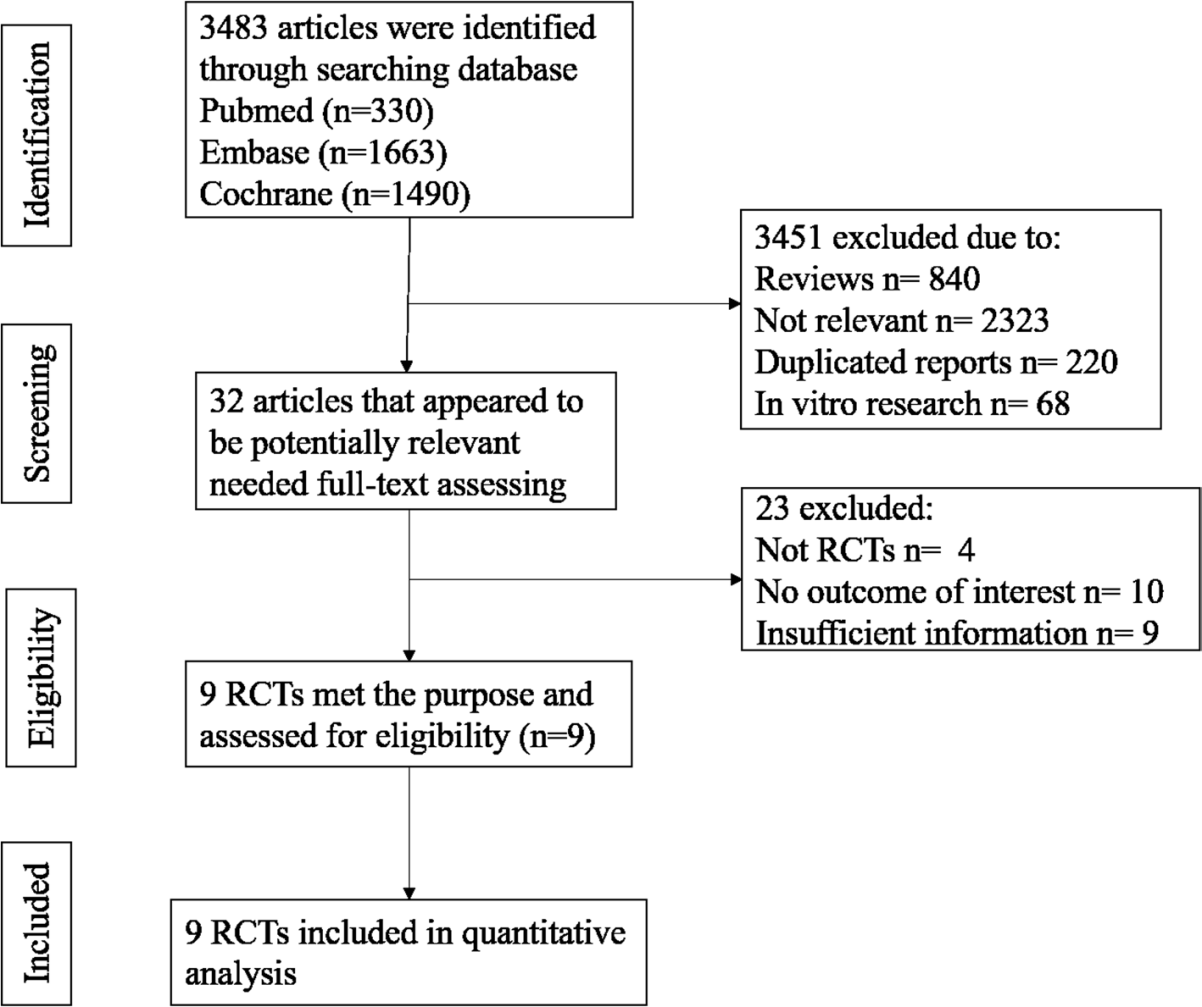
Flow chart of the exclusion and inclusion of studies included in this systematic review and meta-analysis

In total, 3483 articles were identified in the initial search from the database. After checking the titles and abstracts, 3451 articles were excluded due to duplicated articles, not relevant, reviews, case reports and in vitro basic research. Among the 32 articles, 23 articles were excluded due to not RCTs, no outcome of interest and insufficient information after reviewing the full text. Nine studies containing a total of 3098 patients met the including criteria finally. All the included trials evaluated and compared the efficacy and safety of adjuvant EGFR-TKIs with placebo or chemotherapy in patients with resected NSCLC. Among the nine studies, one involved osimertinib, two involved icotinib, four involved gefitinib and two involved erlotinib. In addition, four studies included patients with stage IB to IIIA, two studies included stage II to IIIA, one included stage II to III and the other two included patients with stage IIIA. Furthermore, seven studies had data from patients harboring *EGFR* mutations only and two studies were involved regardless of the *EGFR* mutations status. The flow chart of the study retrieval and data selection was displayed in Fig. 1. The basic characteristics of the included studies was summarized in Table 1.

**Table 1:** Characteristics of the eligible clinical trials

Author and Year	Study phase	Country	Study design	Stage	N	EGFRm+ (%)	Adjuvant CT (%)	Intervention		Duration (months)	Endpoints	Follow-up (months)	mDFS (months)	DFS rate	DFS HR (95% CI)	OS HR (95% CI)
								N	Regimes							
*Tada* 2022 (IMPACT)	Phase 3	Japan	RCT	II-III	232	100%	0%	116	Gefitinib	24	DFS	70	35.9	31.8%(5y)	0.92 (0.67–1.28)	1.03 (0.65–1.65)
						100%	100%	116	NP				25.1	34.1%(5y)		
*He* 2021 (EVIDENCE)	Phase 3	China	RCT	II-IIIA	283	100%	0%	151	Icotinib	22.2	DFS	24.9	47	63.9%(3y)	0.36 (0.24–0.55)	0.91 (0.42–1.94)
						100%	100%	132	NP/PP				22.1	32.5%(3y)		
*Wu* 2020 (ADAURA)	Phase 3	International	RCT	IB-IIIA	682	100%	76% (II-IIA), 26%(IB)	339	Osimertnib	36	DFS in II-IIIA	22.1	NR	90%(2y)	0.17 (0.11–0.26)	0.40 (0.09–1.83)
						100%		343	Placebo				19.6	44%(2y)		
*Zhong* 2018/2021 (ADJUVANT)	Phase 3	China	RCT	II-IIIA	222	100%	0%	111	Gefitinib	24	DFS, OS	80.0	30.8	39.6%(3y) 22.6%(5y)	0.56 (0.40–0.79)	0.92 (0.62–1.36)
						100%	100%	111	NP				19.8	32.5%(3y) 23.2%(5y)		
*Yue* 2018 (EVAN)	Phase 2	China	RCT	IIIA	102	100%	0%	51	Erlotinib	24	DFS, OS	33	42.4	81.4%(2y) 54.2%(3y)	0.27 (0.14–0.53)	0.165 (0.047–0.579)
						100%	100%	51	NP				21	44.6%(2y) 19.8%(3y)		
*Kelly* 2015 (RADIANT)	Phase 3	International	RCT	IB-IIIA express EGFR	973	16.4%	50.6%	623	Erlotinib	24	DFS, OS in ITT	47	50.5	67.2%(2y)	0.90 (0.74–1.10)	1.13 (0.881–1.448)
						16.9%	57.1%	350	Placebo				48.2	62.4%(2y)		
					161	100%	45.1%	102	Erlotinib	24	DFS, OS in EGFRm+	47	46.4	75%(2y)	0.61 (0.384–0.981)	1.09 (0.555–2.161)
						100%	55.9%	59	Placebo				28.5	54%(2y)		
*Feng* 2015	NA	China	RCT	IB-IIIA EGFRm+	41	100%	100%	21	PTX + NDP/LP + Icotinib	4–8	DFS	24	NA	90.5%(2y)	0.45 (0.05–3.81)	NA
						100%	100%	20	PTX + NDP/LP					66.7%(2y)		
*Li* 2014	Phase 2	China	RCT	IIIA-N2	60	100%	100%	30	PC + gefitinib	6	DFS, OS	30.6	39.8	78.9%(2y)	0.37 (0.16–0.85)	0.37 (0.12–1.11)
						100%	100%	30	PC				27	54.2%(2y)		
*Goss* 2013 (NCIC CTGBR19)	Phase 3	North American	RCT	IB-IIIA	503	4%	17%	251	Gefitinib	24	OS, DFS	4.7 years	4.2 years	50.2%(4y)	1.22 (0.93–1.61); 1.84 (0.44–7.73) (EGFRm+)	1.24 (0.94–1.64); 3.16 (0.61–16.45) EGFRm+
						4.3%	17%	252	Placebo				NR	57.6%(4y)		

*NP* Vinorelbine + Cisplatin, *PP* Pemetrexed + Cisplatin, *PTX* Paclitaxel, *NDP* Nedaplatin, *LP* Lobaplatin, *PC* Pemetrexed + carboplatin, *NA* Not available, *NR* Not reached, *N* Number, *CT* Chemotherapy, *RCT* Randomized controlled trial, *DFS* Disease-free survival, *mDFS* Median disease-free survival, *OS* Overall survival, *EGFRm*+: Epidermal growth factor receptor mutation positive, *ITT* Intent-to-treat, *HR* Hazard ratio, *CI* Confidence interval

**Fig. 2:**
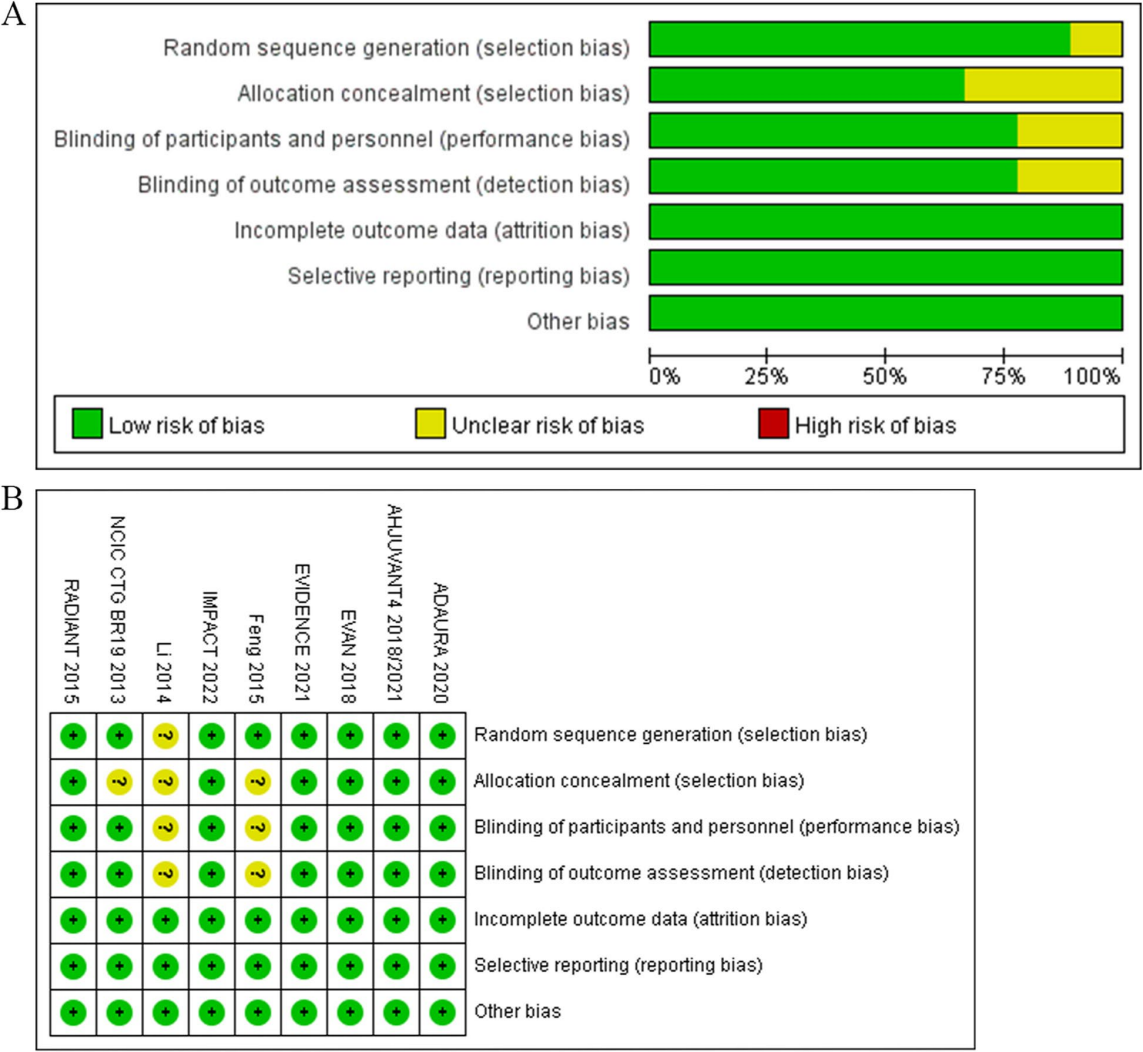
Risk of bias based on the evaluation elements listed in the Cochrane Collaboration Risk of Bias Tool: risk of bias graph (A), risk of bias summary (B)

The quality of the included studies was evaluated by using the Cochrane risk of bias tool. The contents of the risk of bias for each study were presented in Fig. 2. In all, the quality of the trials was satisfactory except a lack of report of HR and 95% CIs of DFS in *Feng* et al. study [19]. The data of *Feng* et al. study was captured and extracted by using a software named Engauge Digitizer and the HR and the 95% CIs of DFS was evaluated by using the method according to the Jayne F Tierney’s introduction [33]. The combined effect of DFS in our meta-analysis was calculated by whether adding *Feng’s* trial or not.

### Effects of adjuvant EGFR-TKIs versus adjuvant chemotherapy/placebo on DFS

**Fig. 3:**
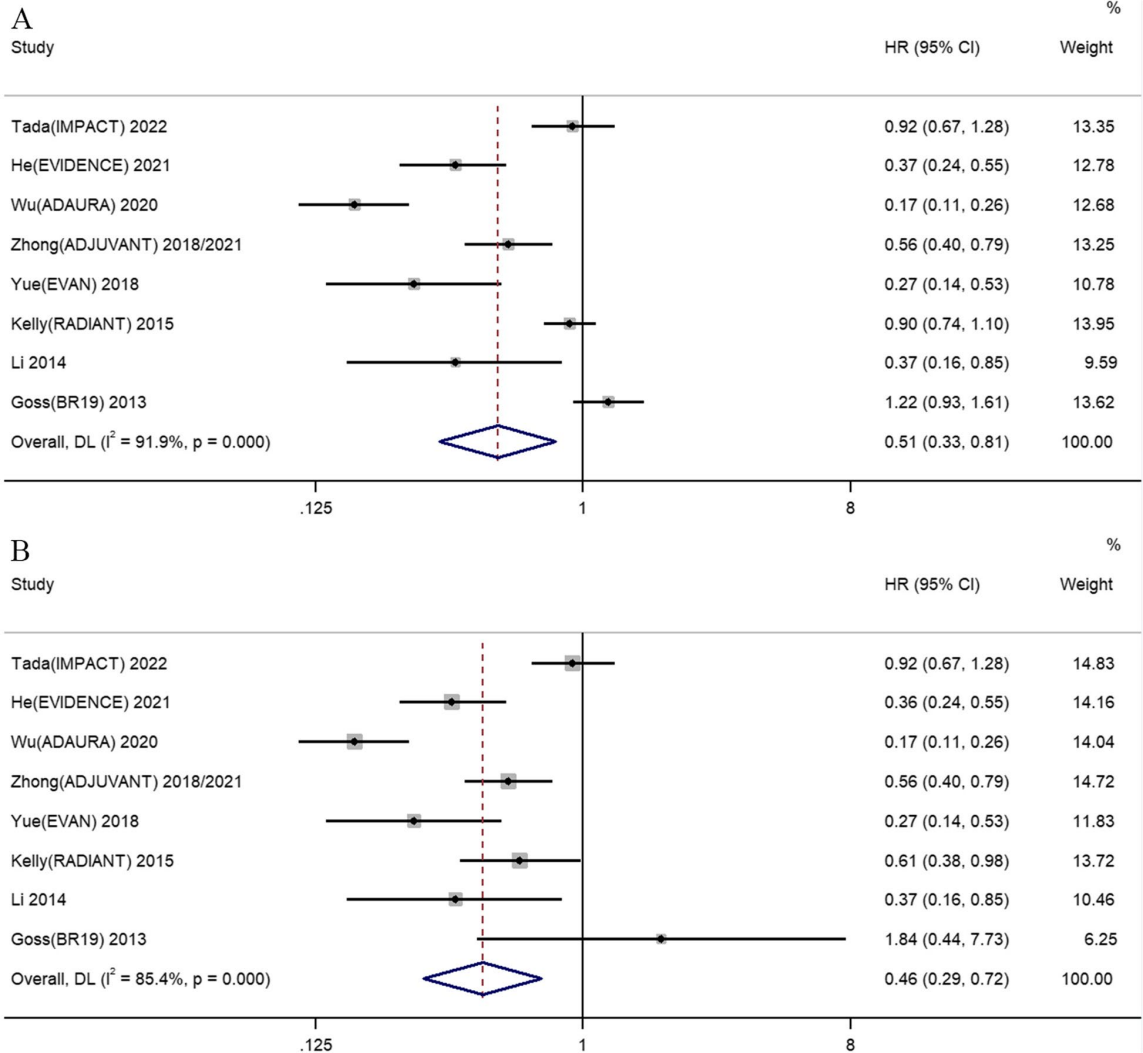
Comparison of DFS between adjuvant EGFR-TKIs versus adjuvant chemotherapy/placebo in resected NSCLC patients. **A** DFS for the intent-to-treat patients with regardless of the *EGFR* mutations status. **B** DFS for patients harboring *EGFR* mutations

As shown in Fig. 3A, eight RCTs reported the data of HR and 95% CI for DFS following adjuvant EGFR-TKIs versus placebo or adjuvant chemotherapy in patients with resected NSCLC. There was significant heterogeneity among the studies, so random-effects statistical models were conducted (I^2^ = 91.9%, *P* = 0.000 and I^2^ = 85.4%, *P* = 0.000 respectively). Our meta-analysis demonstrated that adjuvant EGFR-TKIs could significantly prolonged DFS compared to control group in the intent-to-treat patients with resected NSCLC regardless of the *EGFR* mutations status (HR 0.51, 95% CI 0.33–0.81). The benefit of adjuvant EGFR-TKIs upon DFS was also significant when involving *Feng*’s study (HR 0.51, 95% CI 0.33–0.79) (See Additional file 1). As shown in Fig. 3B, the effect of adjuvant EGFR-TKIs in resected NSCLC patients harboring *EGFR* mutations was further analyzed. The combined results indicated that adjuvant EGFR-TKIs could significantly increase DFS compared to control group in resected NSCLC patients harboring *EGFR* mutations (HR 0.46, 95% CI 0.29–0.72). The effect of adjuvant EGFR-TKIs on DFS was also beneficial when involving *Feng*’s study (HR 0.46, 95% CI 0.29–0.71) (See Additional file 1). We further analyzed the effect of adjuvant EGFR-TKIs versus different control subgroup on DFS (See Additional file 2). The results showed that adjuvant EGFR-TKIs had significant DFS benefit when compared with adjuvant chemotherapy alone (HR 0.50, 95% CI 0.30–0.82). Adjuvant chemotherapy plus EGFR-TKIs was superior in regard to DFS than adjuvant chemotherapy (HR 0.38, 95% CI 0.17–0.83). There was no different between adjuvant EGFR-TKIs and adjuvant placebo group on DFS (HR 0.50, 95% CI 0.15–1.59).

### Effects of adjuvant EGFR-TKIs versus adjuvant chemotherapy/placebo on OS

**Fig. 4:**
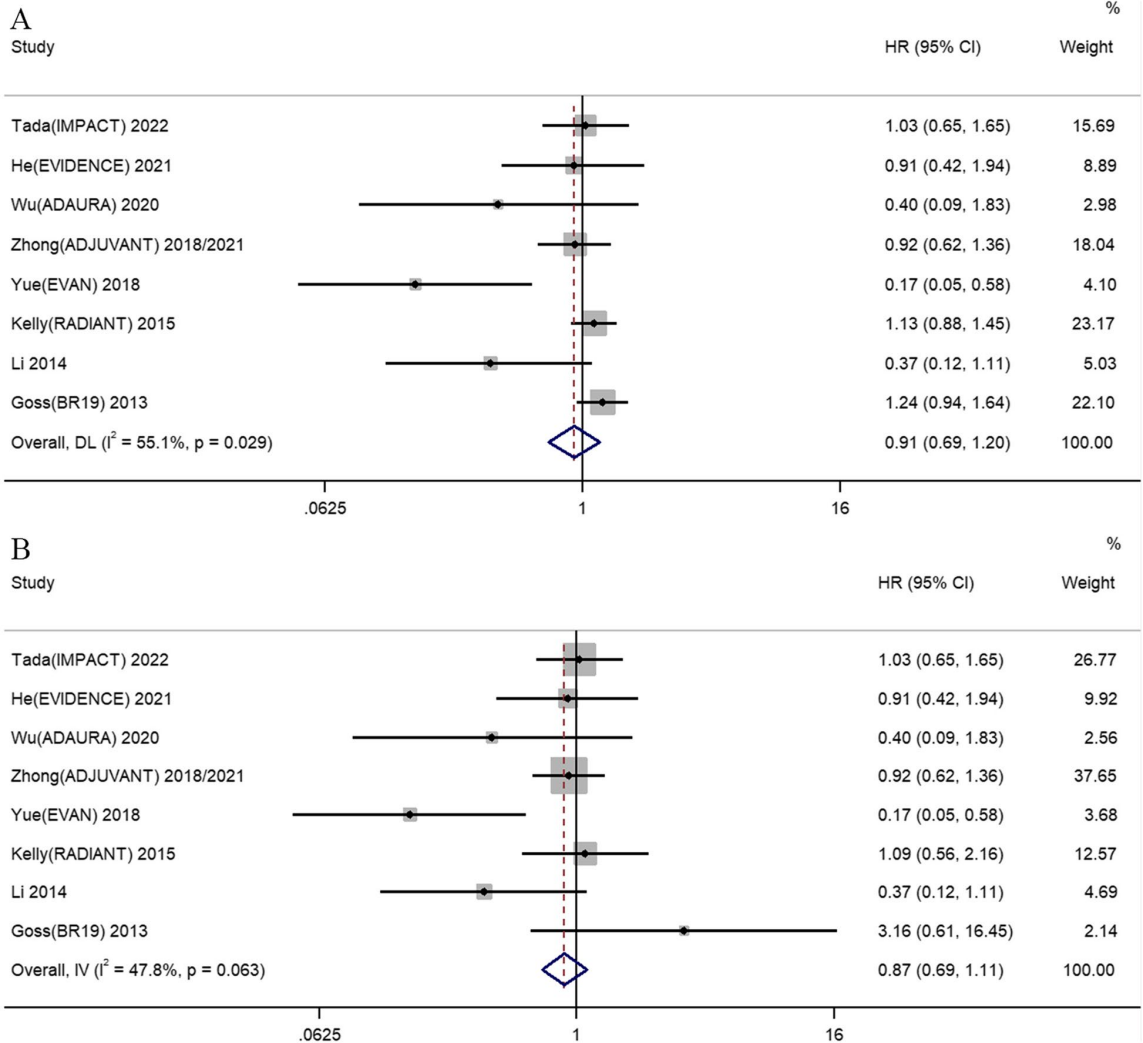
Comparison of OS between adjuvant EGFR-TKIs versus adjuvant chemotherapy/placebo in resected NSCLC patients. **A** OS for the intent-to-treat patients with regardless of the *EGFR* mutations status. **B** OS for patients harboring *EGFR* mutations

As shown in Fig. 4, eight RCTs reported the data of HR and 95% CI for OS following adjuvant EGFR-TKIs versus adjuvant placebo or adjuvant chemotherapy. Our meta-analysis demonstrated that adjuvant EGFR-TKIs had no impact on OS compared to placebo or adjuvant chemotherapy in the intent-to-treat patients with resected NSCLC regardless of the *EGFR* mutations status (HR 0.91, 95% CI 0.69–1.20). There was also no significant increase on OS for adjuvant EGFR-TKIs in NSCLC patients harboring *EGFR* mutations (HR 0.87, 95% CI 0.69–1.11). The subgroup analysis with respect to control group demonstrated that adjuvant EGFR-TKIs had no OS benefit when compared with adjuvant chemotherapy (HR 0.88, 95% CI 0.67–1.16) or adjuvant placebo group (HR 1.07, 95% CI 0.60–1.91). Adjuvant chemotherapy plus EGFR-TKIs was not superior in regard to OS than adjuvant chemotherapy (HR 0.37, 95% CI 0.12–1.13) (See Additional file 2).

### The subgroup analyses of the effects of adjuvant EGFR-TKIs versus adjuvant chemotherapy/placebo on DFS

**Fig. 5:**
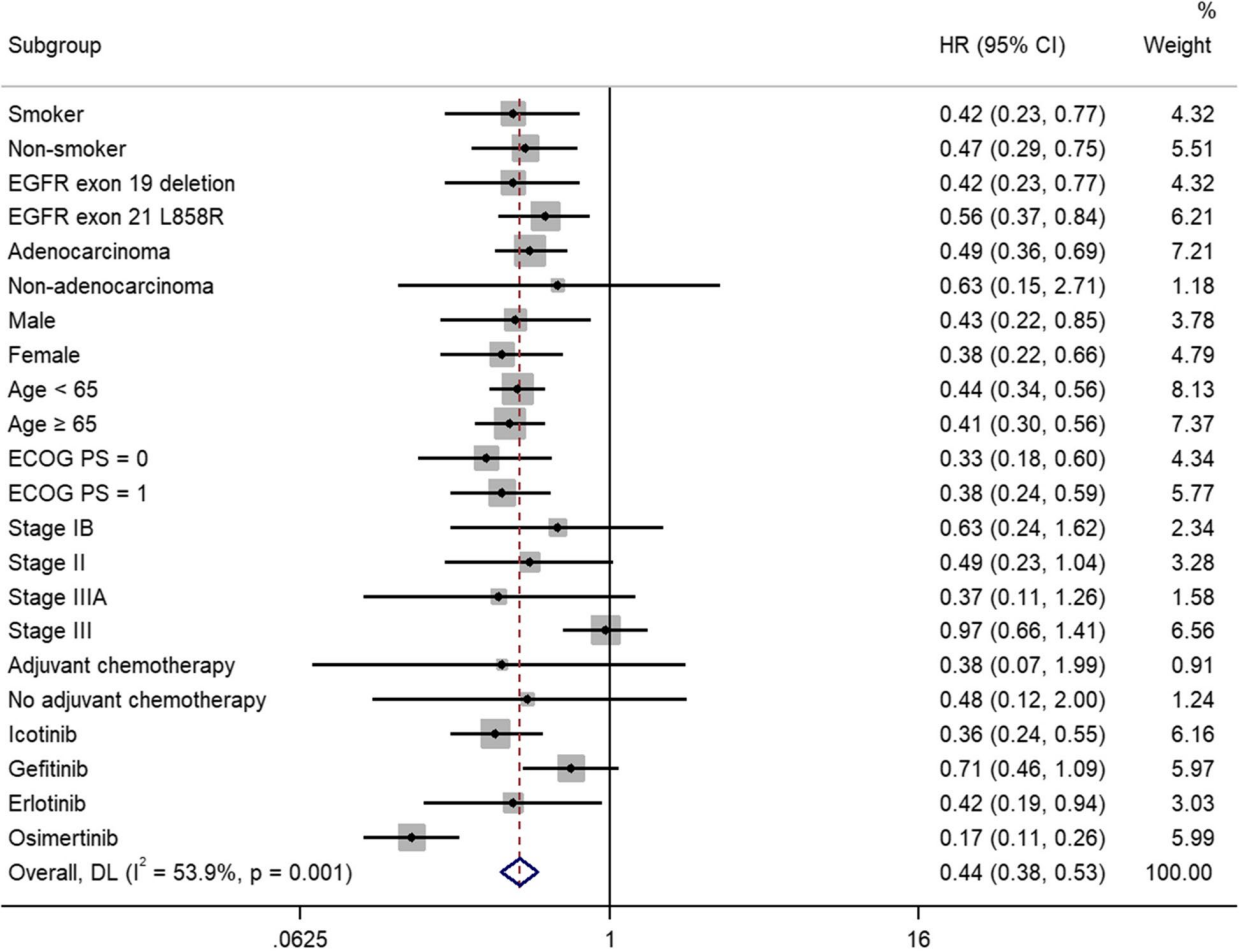
Comparison of DFS between adjuvant EGFR-TKIs versus adjuvant chemotherapy/placebo in the subgroup

As shown in Fig. 5, the effects of adjuvant EGFR-TKIs on DFS were analyzed in the subgroup of smoking status, *EGFR* mutations type, histology, gender, age, ECOG performance status, stage and adjuvant chemotherapy. Our subgroup meta-analyses demonstrated that the benefit of adjuvant EGFR-TKIs over control group with respect to DFS were evident in most subgroups, including smoker (HR 0.42, 95% CI 0.23–0.77), non-smoker (HR 0.47, 95% CI 0.29–0.75), *EGFR* exon 19 deletion (HR 0.42, 95% CI 0.23–0.77), *EGFR* exon 21 L858R (HR 0.56, 95% CI 0.37–0.84), adenocarcinoma (HR 0.49, 95% CI 0.36–0.69), male (HR 0.43, 95% CI 0.22–0.85), female (HR 0.38, 95% CI 0.22–0.66), age < 65 (HR 0.44, 95% CI 0.34–0.56), age ≥ 65 (HR 0.41, 95% CI 0.30–0.56), ECOG was 0 (HR 0.33, 95% CI 0.18–0.60) and 1 (HR 0.38, 95% CI 0.24–0.59). The DFS was not improved in patients with non-adenocarcinoma (HR 0.63, 95% CI 0.15–2.71), stage IB (HR 0.63, 95% CI 0.24–1.62), stage II (HR 0.49, 95% CI 0.23–1.04), stage IIIA (HR 0.37, 95% CI 0.11–1.26), stage III (HR 0.97, 95% CI 0.66–1.41), receiving adjuvant chemotherapy (HR 0.38, 95% CI 0.07–1.99) or not (HR 0.48, 95% CI 0.12–2.00). Additionally, the results indicated that icotinib (HR 0.36, 95% CI 0.24–0.55), osimertinib (HR 0.17, 95% CI 0.11–0.26), and erlotinib (HR 0.42, 95% CI 0.19–0.94) could significantly prolong DFS compared to placebo or adjuvant chemotherapy in the NSCLC patients with *EGFR* mutations. However, the benefit was not presented in patients who receiving gefitinib (HR 0.71, 95% CI 0.46–1.09).

### Effect of adjuvant EGFR-TKIs on disease recurrence

**Fig. 6:**
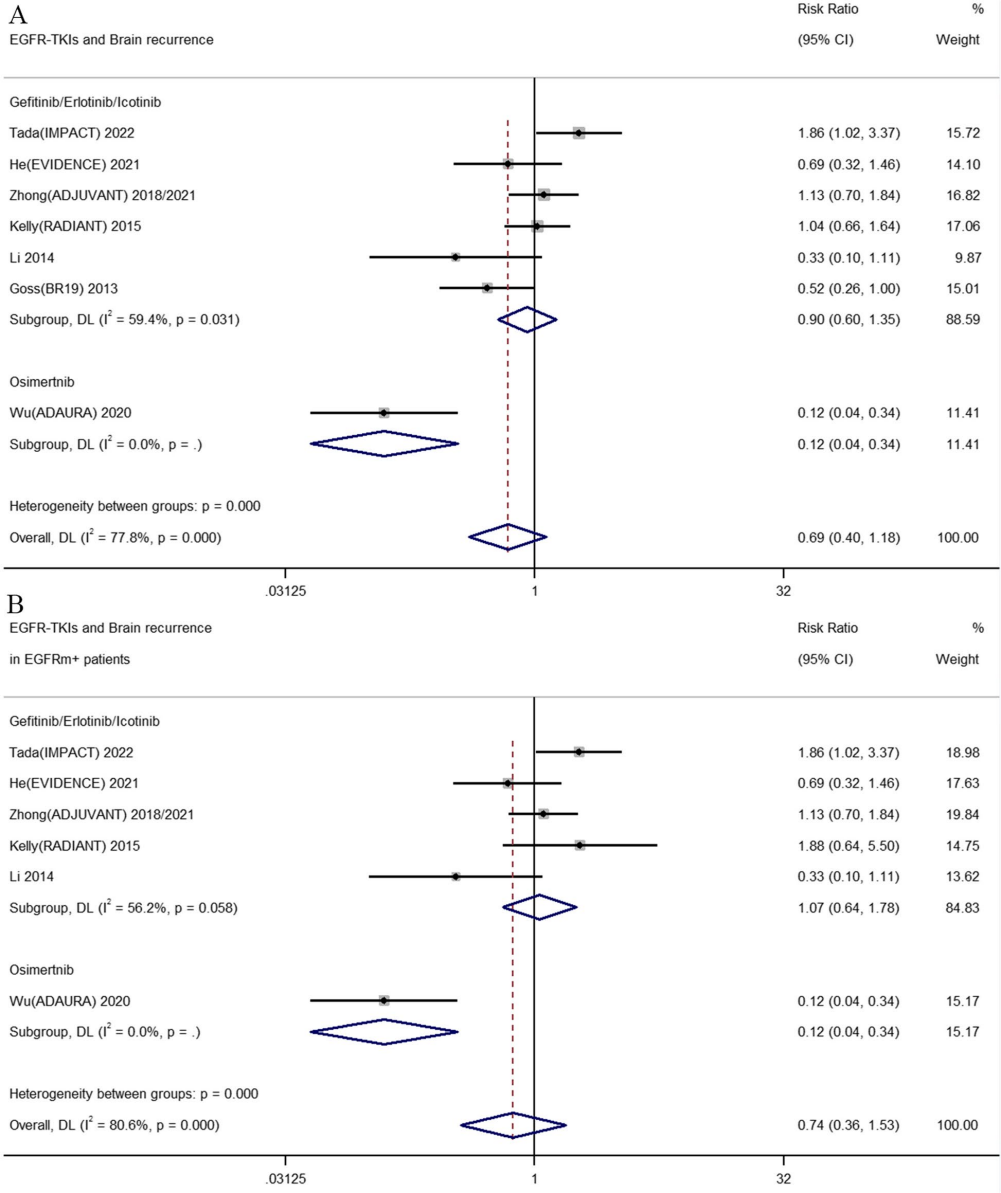
Comparison of brain recurrence between adjuvant first or third-generation of EGFR-TKIs versus adjuvant chemotherapy/placebo. **A** The risk of brain recurrence in intent-to-treat patients with regardless of the *EGFR* mutations status. **B** The risk of brain recurrence in patients harboring *EGFR* mutations

In our meta-analysis, the incidence of relapse was evaluated and analyzed in patients treated with adjuvant EGFR-TKIs versus placebo or adjuvant chemotherapy. The data of disease relapse was available and reported in IMPACT, EVIDENCE ADAURA, ADJUVANT, RADIANT, BR19 and Li et al. trials. In general, the combined results indicated that for local recurrence, distant metastasis, brain, bone, lung, liver, pleural effusion relapse and regional lymph node recurrences, no significant differences were observed between the two groups (See Additional file 3). However, subgroup analysis showed that the incidence of brain recurrence significantly decreased in patients treated with osimertinib (RR 0.12, 95% CI 0.04–0.34) than the first generation of EGFR-TKIs of gefitinib, erlotinib and icotinib (RR 0.90, 95% CI 0.60–1.35) when compared with control group. The result was consistent with the findings in NSCLC patients with *EGFR* mutations (RR 0.12, 95% CI 0.04–0.34 and RR 1.07, 95% CI 0.64–1.78, separately). The brain recurrence rate in IMPACT, EVIDENCE, ADAURA, ADJUVANT, RADIANT, BR19 and Li et al. studies was 22.4, 7 1.2, 27.4, 7.7, 10 and 9.9% in patents receiving adjuvant EGFR-TKIs therapies, respectively. All the related results were shown in Fig. 6 and Additional file 3.

### Safety outcomes

**Fig. 7:**
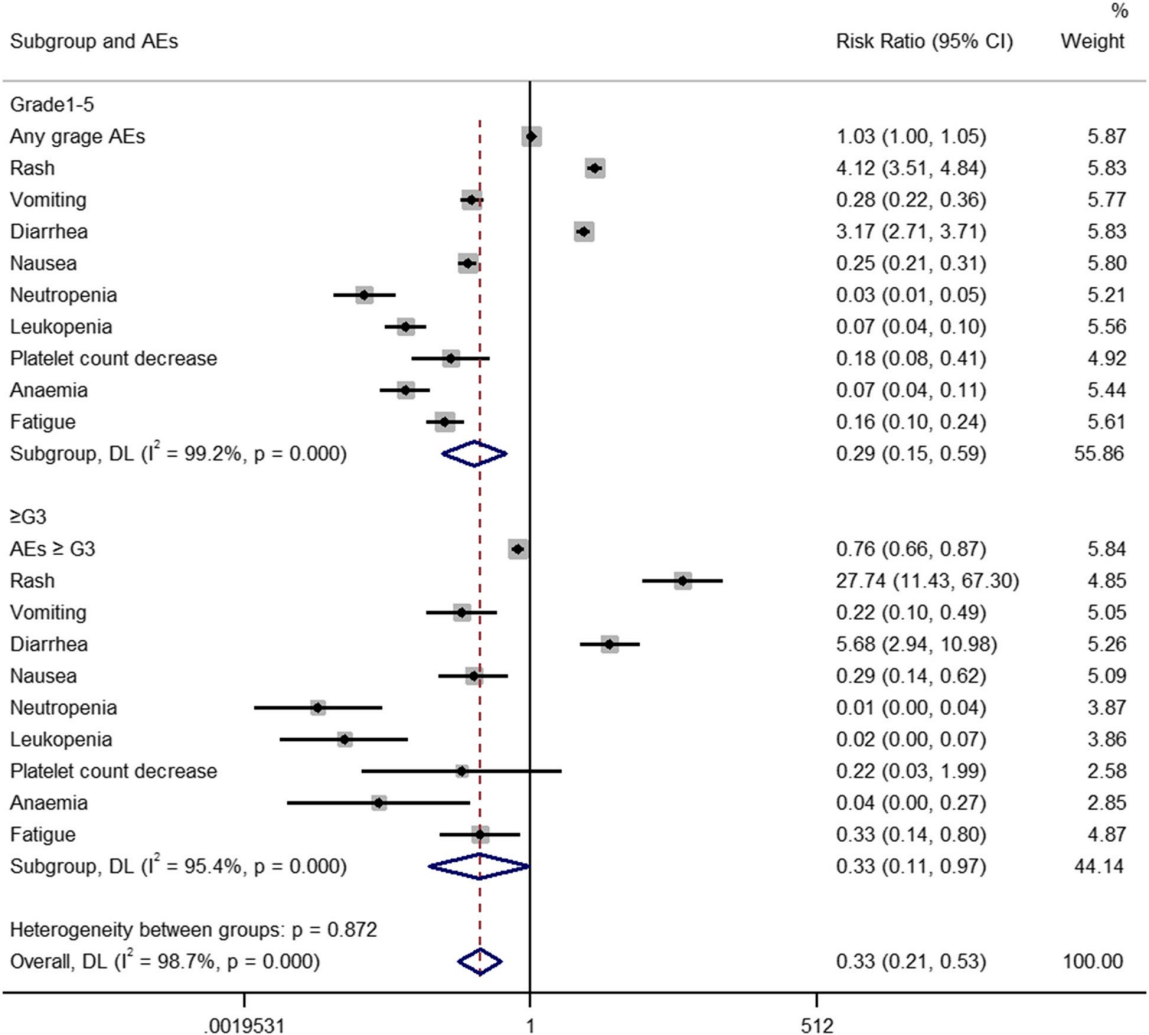
Comparison of RRs of grade 1–5 AEs and grade ≥ 3 AEs between adjuvant EGFR-TKIs versus adjuvant chemotherapy/placebo

The safety outcomes were evaluated and all these adverse events were generally tolerable. As shown in Fig. 7, when compared with the control group, patients treated with adjuvant EGFR-TKIs had lower risk of grade 1–5 AEs (RR 0.29, 95% CI 0.15–0.59). However, the risk of grade 1–5 diarrhea and rash significantly increased in adjuvant EGFR-TKIs group (Diarrhea: RR 3.17, 95% CI 2.71–3.71; Rash: RR 4.12, 95% CI 3.51–4.84). The risk of grade 1–5 vomiting, nausea, neutropenia, leucopenia, platelet count decrease, anaemia and fatigue significantly increased in control group when compared with adjuvant EGFR-TKIs. For high-grade adverse events, patients treated with adjuvant EGFR-TKIs had lower risk of all grade 3–5 AEs (RR 0.33, 95% CI 0.11–0.97). However, the pooled RRs of grade 3–5 diarrhea and rash incidence were significant for the adjuvant EGFR-TKIs group (Diarrhea: RR 5.68, 95% CI 2.94–10.98; Rash: RR 27.74, 95% CI 11.43–67.30). The pooled RRs for grade 3–5 vomiting, nausea, neutropenia, leukopenia, anaemia and fatigue incidence were significant in control group. All the related results in regard to the use of EGFR-TKIs versus control group in resected NSCLC were showed in Fig. 7.

### Publication bias

The *P* value was 0.902 for Begg’s test and 0.734 for Egger’s test. Therefore, there was no evidence of publication bias for DFS in this meta-analysis (*P* > 0.05).

## Discussion

In our current meta-analysis, the results showed that adjuvant EGFR-TKIs could significantly prolong DFS in patients with resected early-stage *EGFR* mutations-positive NSCLC, with manageable and tolerable toxicity. However, no significant difference with respect to OS improvement was observed. Besides, the treatment failure patterns in terms of relapse places were analyzed and the results demonstrated that brain recurrence was significantly lower in patients with adjuvant osimertinib than with the first generation of EGFR-TKIs of gefitinib, erlotinib and icotinib.

To our knowledge, whether EGFR-TKIs could be an adjuvant treatment in operable NSCLC patients had been a controversial subject for decades. Several studies had reported conflicting results on the clinical effect of adjuvant EGFR-TKIs in resected NSCLC in the past and in recent years [6, 17–22]. IMPACT, RADIANT, BR19 and *Feng* et al. studies demonstrated that no survival benefits were observed in resected NSCLC patients with adjuvant erlotinib, gefitinib or icotinib [17, 19, 20, 28]. Whereas the DFS with adjuvant gefitinib in ADJUVANT and Li et al. trials and erlotinib in EVAN trial was significantly improved [18, 22]. It was worth noted that the DFS was prolonged with statistically significant in patients with *EGFR* mutations in RADIANT trial [20]. Moreover, the recent studies of ADAURA and EVIDENCE showed that adjuvant osimertinib or icotinib could provide significant survival benefits against placebo or adjuvant chemotherapy in NSCLC patients with *EGFR* mutations [23, 24]. The reasons for the contradictory results might be caused, at least in part, by that as follows: (1) there was apparent inconsistency in clinical stage; (2) the percentages of *EGFR* mutations were different, which varied from 4% in BR19 to 100% in most of studies; (3) different generation of EGFR-TKIs was chosen as intervention therapies; (4) the duration of EGFR-TKIs was non-uniform. (5) the timing with respect to medication was inconsistent.

Prior meta-analysis had noted the importance of adjuvant EGFR-TKIs on DFS benefit in resected NSCLC [25–27, 34, 35]. The present updated meta-analysis showed that adjuvant EGFR-TKIs could improve the DFS of resected early-stage NSCLC harboring *EGFR* mutations, when compared with the control group. Whereas only osimertinib is recommended historically initially as a considerable adjuvant treatment followed adjuvant chemotherapy by NCCN guideline, and none of the other EGFR-TKIs are recommended by guideline [29]. The reason might be that although the DFS benefits of first generation of EGFR-TKIs were significant in EVIDENCE, ADJUVANT, EVAN and other studies, the DFS advantage associated with adjuvant icotinib, gefitinib and erlotinib did not translate into a significant OS improvement [21, 22, 24]. However, in ADAURA study, the median DFS was not reached in adjuvant osimertinib versus 27.5 months in placebo group [23]. The statistically significant HR for DFS was as low as 0.20 in resected NSCLC with stage IB-IIIA, and was 0.17 in stage II-IIIA patients, which was the historic lows in adjuvant therapies [23]. Even though the OS data was immature, 98% of the patients in the osimertinib and 85% of those in the placebo group were alive without central nervous system (CNS) metastasis at 24 months and the median CNS DFS was not reached and 48.2 months, respectively. Additionally, it also couldn’t be ignored that icotinib achieved an impressive median DFS of 47 months in EVIDENCE trial and erlotinib achieved 42.4 months in EVAN trial compared to adjuvant chemotherapies [21, 24]. They might be alternative adjuvant therapies in some certain occasions in clinic.

Despite improvements in DFS, adjuvant EGFR-TKIs versus chemotherapy or placebo had not substantially improved OS in patients with operable NSCLC, not matter what the *EGFR* mutations status were. The final OS could be affected by many factors. The subsequent treatment options, such as chemotherapy, radiation, immunotherapy, operation, continuing the same EGFR-TKIs or changing to another generation of EGFR-TKIs, the best supportive cere or wait and watch, were markedly different between individuals after disease recurrence or metastasis [36]. The final median OS had no significant difference between gefitinib and vinorelbine plus cisplatin group in the ADJUVANT trial (median OS: 75.5 months versus 62.8 months; HR 0.92, 95% CI 0.62–1.36; *P* = 0.574, 36]. The updated data showed that subsequent therapies greatly influenced OS and continuing EGFR-TKIs contributed most to OS in ADJUVANT trial. In addition, the duration of EGFR-TKIs might also influenced the prognosis. The average duration of adjuvant EGFR-TKIs was 24 months in most of the clinical trials [17, 20–22]. In the ADAURA trial, adjuvant osimertinib medication was required for up to 36 months [23, 29]. So, the point is, whether a longer maintenance treatment with EGFR-TKIs can further improve the OS remains unclear. The subsequent final OS data in ADAURA trial is anticipated and more investigations are urgently needed.

Subgroup analysis demonstrated that the risk of brain recurrence was significant reduced when patients treated with adjuvant osimertinib than with first generation of EGFR-TKIs. Why osimertinib could reduce the rate of brain metastasis? First, osimertinib was a potent and irreversible inhibitor of *EGFR* kinase activity and was structurally different from other generations of TKI [37]. Second, the preclinical study revealed that it can suppress the growth of cells in both *EGFR* sensitizing mutations and *EGFR* second mutation cell lines, which resulted in substitution of threonine at amino acid 790 to methionine (T790M), in vitro [38]. In addition, the preclinical data displayed osimertinib demonstrated better penetration of blood-brain barrier, achieved brain exposure and high distribution in central nervous system, and could cause sustained disease remission than gefitinib, rociletinib, or afatinib in mouse xenograft models harboring *EGFR* mutations [38]. On the basis of the excellent outcomes of osimertinib from previous studies, it was approved as a second-line and first-line treatment for patients with metastatic *EGFR* sensitizing mutations and T790M positive NSCLC, subsequently [39, 40]. The longer median progressive-free survival in CNS metastases patients treated with osimertinib than first-generation EGFR-TKIs was also observed and reported [12]**.**

However, there still remains a few problems in the treatment of adjuvant EGFR-TKI*s*. First, the final goal of adjuvant treatment was to improve the OS, but DFS rather than OS was designed as the primary endpoint in most clinical trials. Although DFS could be applied as a surrogate of OS for NSCLC, this pattern was mainly existed in studies treated with chemotherapies in which DFS was consistent with OS [41]. However, this situation was different in adjuvant EGFR-TKIs treatment patterns. Second, whether adjuvant EGFR-TKIs can lead to tumor resistance untimely and develop complex resistance mechanisms that without effective drugs in the absence of bulky tumor burden remains unclear. When the disease progress after adjuvant EGFR-TKIs in resected NSCLC, how the illness should be handled? Whether continuing the same EGFR-TKIs or other generations of EGFR-TKIs as first-line treatment can gain the equivalent efficacy, when compared with first usage of EGFR-TKIs in metastatic settings? If not, patients will lose a genuine opportunity of receiving the first-line EGFR-TKIs when disease relapses [42]. Third, although adjuvant EGFR-TKIs were considered to be well-tolerated, our analysis indicated that the risks of diarrhea and rash were significantly higher. Therefore, if long-term survival benefits were not unequivocal, suffering such side effects for serval years during the medication period seemed no rewarding. Forth, the economic conditions must be considered since it was not easy for most of patients to afford target drugs for long period. To sum up, although adjuvant EGFR-TKI is recommended in operable NSCLC, the situations mentioned above should be fully concerned and overtreatments should be avoided in clinical practice.

Besides, there were some limitations in our analysis. First, the definition of experimental and control groups had a shortcoming that the control group included adjuvant placebo or adjuvant chemotherapy without specific distinguishing. In order to reduce the bias, additional analyses were conducted according to different interventions of experimental and control groups (See Additional file 2). The reasons why adjuvant EGFR-TKIs were not superior to placebo might be as follows: (1) a certain percentage of extra adjuvant chemotherapies were allowed in the placebo group; (2) the proportion of *EGFR* mutations was too low in BR 19 trial which may influenced the result greatly (4 and 4.3%, respectively). Second, both first and third-generation EGFR-TKIs were included in this meta-analysis. The reasons were as follows: (1) both of them belonged to small molecule EGFR-TKIs, aiming at blocking the *EGFR* signal pathway and inhibiting tumor growth; (2) they were all recommended in the first-line treatment of advanced NSCLC harboring *EGFR* mutations; (3) few published articles focused on the effect of third-generation TKIs as adjuvant treatment and the excellent outcome generated from ADAURA trial made us produce the data synthesis in one single analysis. Third, the follow-up was short in most of the included studies. The IMPACT had a median follow-up of 70 months and the result showed adjuvant gefitinib seemed to prevent early relapse than adjuvant cisplatin plus vinorelbine, but the two curves with respect to DFS crossed about 4 years after surgery. Therefore, longer follow-up might represent different outcomes. Forth, most clinical trials excluded patients with uncommon *EGFR* mutations, which harbored heterogeneous molecular alterations within exons 18–21 and collectively accounted for 10% of *EGFR* mutations. Previous studies showed that the clinical efficacy of EGFR-TKIs in advanced NSCLC harboring *EGFR* uncommon mutations was variable and the OS was shorter when compared to classical mutations [43–45]. Moreover, the lack of sufficient high-quality prospective clinical evidence, no firm standard of care and obvious heterogeneity in detection methods had limited the exploration of EGFR-TKIs as adjuvant therapy in NSCLC patients with less common *EGFR* mutations.

## Conclusions

Our up-to-date analysis demonstrated that adjuvant EGFR-TKIs therapy could significantly prolong DFS in patients with resected early-stage NSCLC harboring *EGFR* mutations. However, no OS benefit was observed when compared with placebo or adjuvant chemotherapy in this population. The third-generation EGFR-TKI, osimertinib, was superior in preventing the incidence of brain metastasis than the first-generation of EGFR-TKIs as adjuvant therapy in resected NSCLC. The toxicity of adjuvant EGFR-TKIs therapy was generally manageable and tolerable. Although there were possible shortcomings in the included studies, such as study design deficiency, inconsistency in clinical stage, various EGFR-TKIs included, non-uniform duration of EGFR-TKIs, inconsistent timing of medication and economic condition limiting and so on, the present study still provided an important and relative satisfactory clinical practice options for early-stage resected NSCLC patients harboring *EGFR* mutations.

## Supplementary Information

**Additional file 1 Supplementary Fig. 1.** Comparison of DFS between adjuvant EGFR-TKIs versus adjuvant chemotherapy/placebo in resected NSCLC patients when involving *Feng’s* study. (A) DFS for the intent-to-treat patients with regardless of the *EGFR* mutations status. (B) DFS for patients harboring *EGFR* mutations.

**Additional file 2 Supplementary Fig. 2.** Comparison of DFS and OS among adjuvant EGFR-TKIs versus chemotherapy subgroup, adjuvant EGFR-TKIs versus placebo, adjuvant chemotherapy plus EGFR-TKIs versus adjuvant chemotherapy subgroup. (A) DFS comparison. (B) OS comparison.

**Additional file 3 Supplementary Fig. 3.** Comparison of local recurrence, distant metastasis and other subgroup recurrences between adjuvant EGFR-TKIs versus adjuvant chemotherapy/placebo. (A) Local recurrence. (B) Distant metastasis. (C) The subgroup recurrences (including bone recurrence, lung local recurrence and distant metastasis, liver recurrence, pleural diffusion recurrence and regional lymph node recurrence)

## Data Availability

The datasets used and/or analyzed during the current study are available from the corresponding author on reasonable request.

## Abbreviations

NSCLC: Non-small cell lung cancer
EGFR: Epidermal growth factor receptor
EGFR-TKIs: Epidermal growth factor receptor tyrosine kinase inhibitors
RCTs: Random control trials
HR: Hazard ratio
CI: Confidence intervals
DFS: Disease-free survival
OS: Overall survival
RR: Risk ratio
AEs: Adverse events
ECOG: Eastern Cooperative Oncology Group
NCCN: National Comprehensive Cancer Network
PRISMA: Preferred Reporting Items for Systematic Reviews and Meta-Analyses
CNS: Central nervous system
L858R: Point mutation in exon 21 resulting in substitution of leucine for arginine at position 858
T790M: Substitution of threonine at amino acid 790 to methionine
