# Supplementary figures and images for: Efficacy and safety of adjuvant EGFR-TKIs for resected non-small cell lung cancer: a systematic review and meta-analysis based on randomized control trials
Source: BMC Cancer. 2022 Mar 26;22:328. doi: 10.1186/s12885-022-09444-0 (PMC8962534; doi:10.1186/s12885-022-09444-0)

## Slide 1
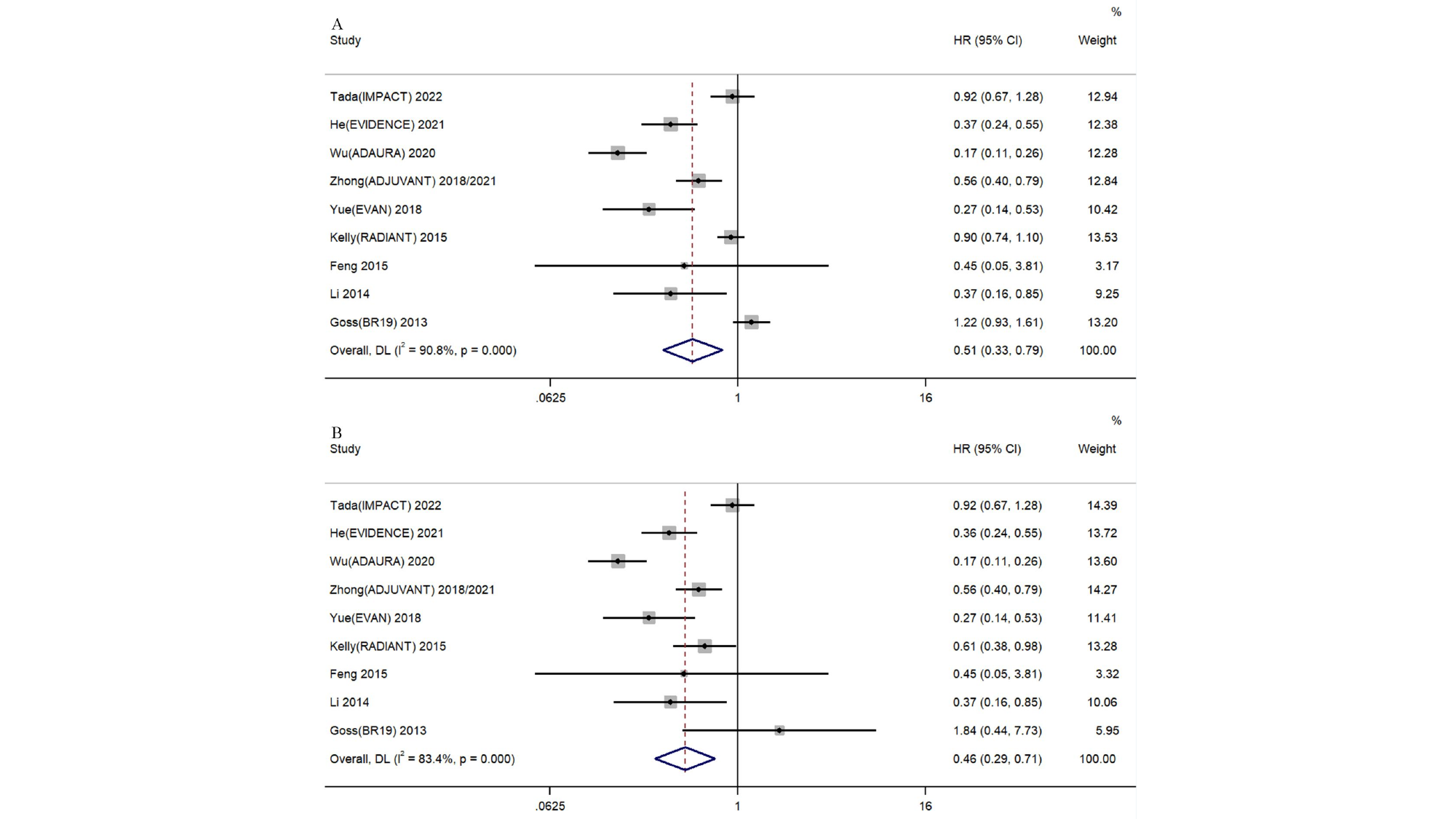

Supplement: Supplementary file 1 — Additional file 1 Supplementary Fig. 1. Comparison of DFS between adjuvant EGFR-TKIs versus adjuvant chemotherapy/placebo in resected NSCLC patients when involving Feng’s study. (A) DFS for the intent-to-treat patients with regardless of the EGFR mutations status. (B) DFS for patients harboring EGFR mutations. [file 12885_2022_9444_MOESM1_ESM.pptx]

## Slide 1
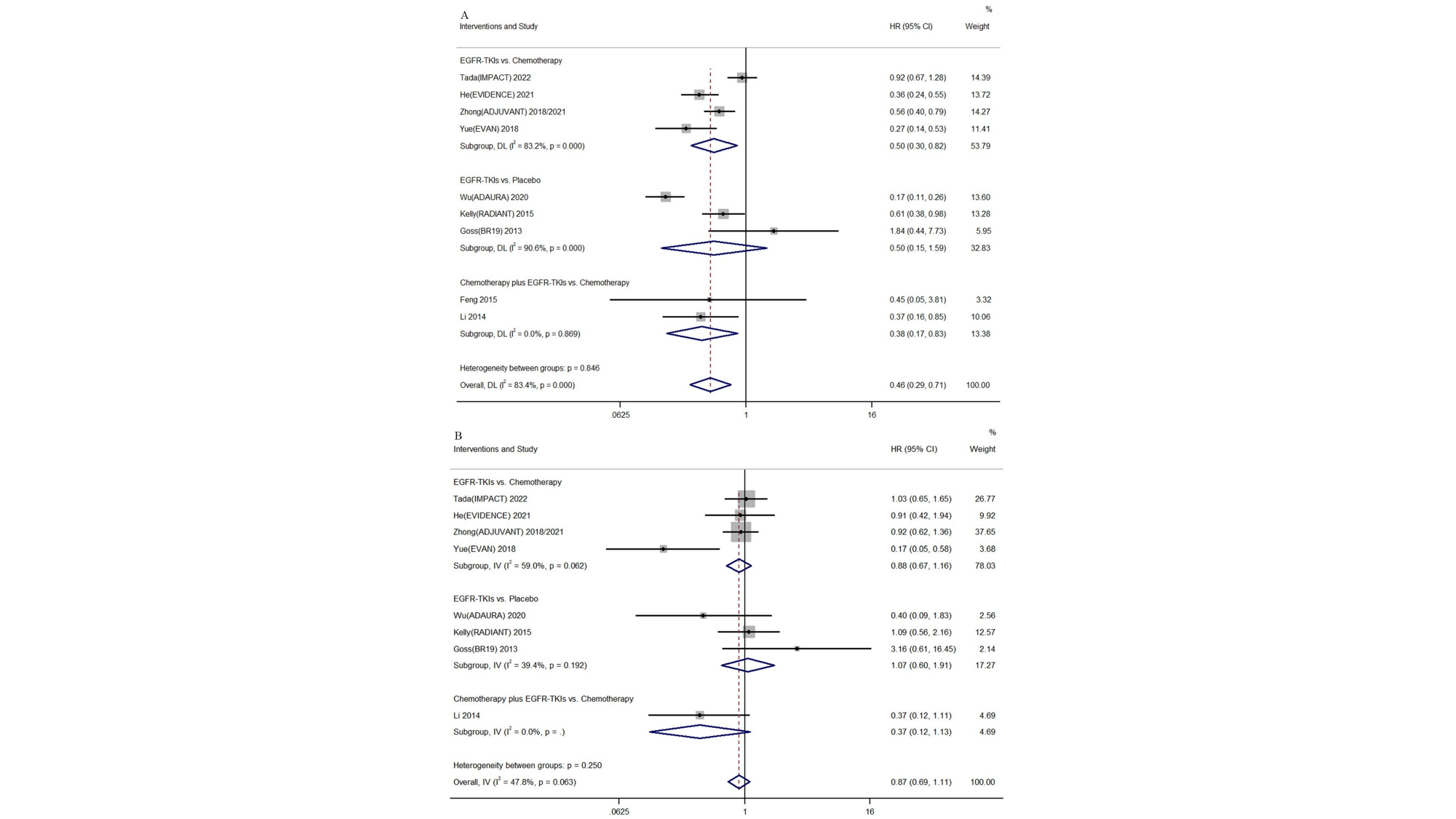

Supplement: Supplementary file 2 — Additional file 2 Supplementary Fig. 2. Comparison of DFS and OS among adjuvant EGFR-TKIs versus chemotherapy subgroup, adjuvant EGFR-TKIs versus placebo, adjuvant chemotherapy plus EGFR-TKIs versus adjuvant chemotherapy subgroup. (A) DFS comparison. (B) OS comparison. [file 12885_2022_9444_MOESM2_ESM.pptx]

## Slide 1
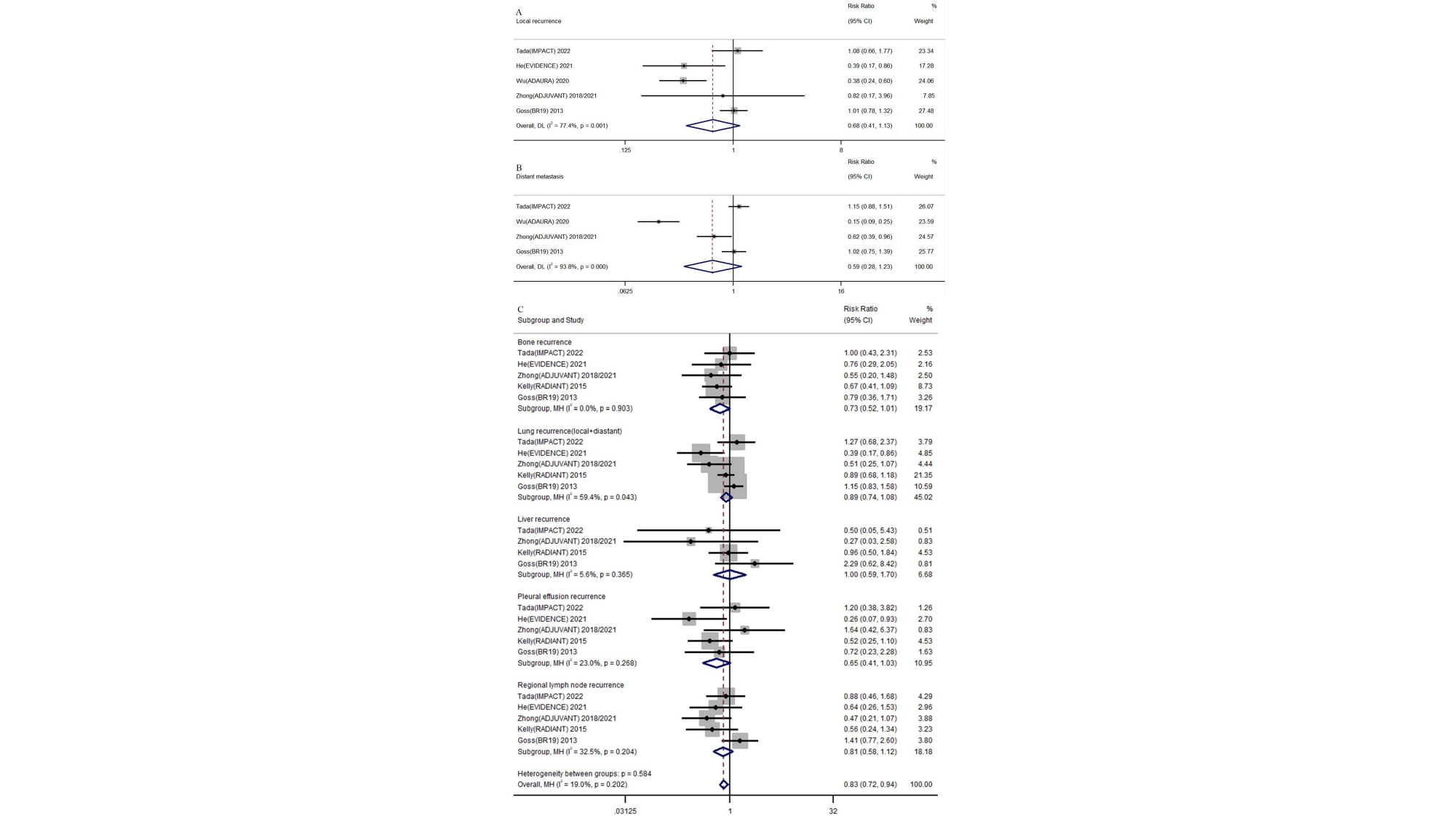

Supplement: Supplementary file 3 — Additional file 3 Supplementary Fig. 3. Comparison of local recurrence, distant metastasis and other subgroup recurrences between adjuvant EGFR-TKIs versus adjuvant chemotherapy/placebo. (A) Local recurrence. (B) Distant metastasis. (C) The subgroup recurrences (including bone recurrence, lung local recurrence and distant metastasis, liver recurrence, pleural diffusion recurrence and regional lymph node recurrence) [file 12885_2022_9444_MOESM3_ESM.pptx]
